# The socioeconomic burden of pediatric tuberculosis and role of child-sensitive social protection

**DOI:** 10.1186/s12889-023-17084-7

**Published:** 2023-11-25

**Authors:** Kinari Shah, Jascent Nakafeero, Jillian L. Kadota, Peter Wambi, Gertrude Nanyonga, Emma Kiconco, Atwiine Deus, Moorine P. Sekadde, Mary G. Nabukenya-Mudiope, Hellen Aanyu- Tukamuhebwa, Ezekiel Mupere, Swomitra Mohanty, Adithya Cattamanchi, Eric Wobudeya, Priya B. Shete, Devan Jaganath

**Affiliations:** 1 Division of Pulmonary and Critical Care Medicine, University of California, San Francisco, San Francisco, CA USA; 2 Center for Tuberculosis, University of California, San Francisco, San Francisco, California USA; 3 Mulago National Referral Hospital, Kampala, Uganda; 4 National TB and Leprosy Program, Ministry of Health, Kampala, Uganda; 5 Infectious Diseases Institute, Kampala, Uganda; 6 Department of Pediatrics and Child Health, Makerere University College of Health Sciences, Kampala, Uganda; 7 Departments of Chemical Engineering and Materials Science Engineering, University of Utah, Salt Lake City, Utah USA; 8 Division of Pulmonary Diseases and Critical Care Medicine, University of California, Irvine, Orange, California USA; 9 Division of Pediatric Infectious Diseases, University of California, San Francisco, California USA

**Keywords:** Child, Cost, Tuberculosis, Social protection

## Abstract

**Background:**

Households of children with tuberculosis (TB) experience financial and social hardships, but TB-specific social protection initiatives primarily focus on adults.

**Methods:**

We conducted a single-arm, pilot study of multi-component supportive benefits for children with pulmonary TB in Kampala, Uganda. At diagnosis, participants received in-kind coverage of direct medical costs, a cash transfer, and patient navigation. Caregivers were surveyed before diagnosis and 2 months into TB treatment on social and financial challenges related to their child’s illness, including estimated costs, loss of income and dissaving practices.

**Results:**

We included 368 children from 321 households. Pre-diagnosis, 80.1% of caregivers reported that their child’s illness negatively impacted household finances, 44.1% of caregivers missed work, and 24% engaged in dissaving practices. Catastrophic costs (> 20% annual income) were experienced by 18.4% (95% CI 13.7–24.0) of households. School disruption was common (25.6%), and 28% of caregivers were concerned their child was falling behind in development. Two months post-diagnosis, 12 households (4.8%) reported being negatively affected by their child’s TB disease (difference -75.2%, 95% CI -81.2 to -69.2, *p* < 0.001), with limited ongoing loss of income (1.6*%*) or dissavings practices (0.8%). Catastrophic costs occurred in one household (0.4%) at 2 months post-diagnosis.

**Conclusions:**

Households face financial and social challenges prior to a child’s TB diagnosis, and child-sensitive social protection support may mitigate ongoing burden.

**Supplementary Information:**

The online version contains supplementary material available at 10.1186/s12889-023-17084-7.

## Introduction

Tuberculosis (TB) disease and its treatment places a large financial burden on individuals and families [1]. Social protection interventions, such as cash transfers, nutritional supplements, or transport vouchers, can reduce the negative financial effects of TB and improve treatment outcomes [2–4]. While the World Health Organization (WHO) END TB strategy has called for increased social protection measures [5], 48% of people with TB still had catastrophic costs globally in 2022 [6].

Current social protection interventions focus on supporting adults with TB [7], but programs for households of children with TB are lacking. Children are more than twice as likely to live in extreme poverty than adults [8], and are more susceptible to TB and other infections due to risk factors including malnutrition and crowding [9, 10]. These shocks can worsen poverty as caregivers face additional costs for evaluation, treatment and follow-up visits, as well as lost income from missed work or childcare needs [11, 12]. Additionally, missed school and developmental delays can perpetuate inter-generational poverty and poor life outcomes [13]. Social protection programs for children can improve growth, education and development [14–16], but there is insufficient data on how these interventions can support TB care for children.

Caregivers have reported that their child’s TB illness adds financial and emotional challenges on their household [17, 18]. Children with TB can have non-specific symptoms and negative sputum testing due to paucibacillary disease [19], delaying TB diagnosis and extending this burden. In Uganda, 23% of children live below the national poverty line [20]. The Uganda national TB costing survey reported that 65% of families of children with TB incurred catastrophic costs [21]. Thus, there is a need to better characterize their challenges and the child-sensitive interventions to mitigate the negative social and financial effects of TB.

We assessed the pre-diagnosis social and financial burden of pediatric TB in Kampala, Uganda, and evaluated changes after families received multi-component benefits inclusive of TB treatment support and social protections at a pediatric TB clinic.

## Methods

### Setting and study population

We assessed changes in socioeconomic burden among children (aged 0–14 years) and their caregivers who received a package of supportive benefits as part of an observational study at Mulago National Referral Hospital Pediatric TB Clinic in Kampala, Uganda [22]. Children were referred from pediatric inpatient wards, outpatient clinics, and other community facilities from Kampala Capital City Authority (KCCA) and the Infectious Diseases Institute (IDI). For this analysis, we included children who were initiated on treatment for drug-susceptible TB by clinic providers as per the national TB guidelines [23], either as microbiologically-confirmed or clinically-diagnosed TB. All participants had new diagnoses of pediatric TB at the initial evaluation. As the goal was to assess pre-diagnosis burden, children were excluded if they were already on TB treatment or were diagnosed and treated for TB in the last year. Children were also excluded if they had drug-resistant TB as care happens at separate dedicated clinics, and families of children with drug-resistant TB also face different financial burdens [18]. All children had follow-up visits per clinic guidelines at two and four weeks for treatment refills and clinical assessment, then monthly visits for treatment refills up to six months. We included children who were enrolled from November 2018 to July 2021. All children were minors, and informed written consent was obtained from a parent and/or legal guardian, and capable children eight years and older completed an assent. The study was approved by the University of California, San Francisco Committee on Human Research, the Mulago Hospital Research and Ethics Committee (MHREC), and the Uganda National Council of Science and Technology.

### Intervention description

At the clinic, participants were provided:

1. *In-kind direct medical costs*. Standard free TB workup in Uganda includes a clinical evaluation, HIV testing, chest x-ray and Xpert MTB/RIF testing (Cepheid, Sunnyvale, USA) if the patient can expectorate sputum. However, due to service scarcities, caregivers must often contribute a fee for certain diagnostic services. This includes approximately 100,000 Uganda Shillings (Ushs) for a two-view chest x-ray, 80,000 Ushs to collect respiratory samples if a child cannot expectorate, and 40,000 Ushs for tuberculin skin testing (TST). In this study, all participants received expanded assessment at no cost. Standard treatment for drug-susceptible TB was provided for free by the Ministry of Health in Uganda.
2. *Cash transfer to reimburse direct non-medical costs*. Caregivers received reimbursements in cash to adequately cover transportation and direct non-medical costs for care-related visits, with the amount guided by similar studies and MHREC. If the child was evaluated as an outpatient, they received 30,000 Ushs per visit, and an additional 10,000 Ushs per visit if the child was 8 years or older to cover the charge for the older child’s seat in the taxi (1 USD =  ~ 3600 Ushs, up to 22 USD given in total for a baseline visit and day 2–3 visit to read the TST). If the child was enrolled from the inpatient wards, caregivers received 15,000 Ushs (4 USD) to provide some direct non-medical cost reimbursement as there were no additional transportation needs.
3. *Patient navigation*. Trained study nurses helped caregivers collect respiratory samples from the child for TB diagnosis, obtain a chest x-ray, and navigate the process to complete diagnostic evaluation and receive TB treatment. This type of patient navigation is not usually part of the standard of care.

### Outcomes

The primary outcome was catastrophic costs due to TB at pre-diagnosis and then 2 months after TB diagnosis and initiation of treatment. Catastrophic costs were defined as total costs exceeding 20% of the household annual income. Secondary outcomes included changes in costs and dissaving practices at pre- and post-diagnosis. Total costs were defined as the combination of direct medical care costs, direct non-medical costs, and indirect costs from loss of income or other opportunities, and were reported as a proportion of annual income. We did not incorporate the cash transfer into the cost calculation at 2 months. Dissaving practices are defined as practices that reduce savings such as selling assets, borrowing, and taking out a loan. Other secondary outcomes included the impact of seeking TB care, such as missing school or perceived developmental delays by caregivers.

### Measurements

**Fig. 1:**
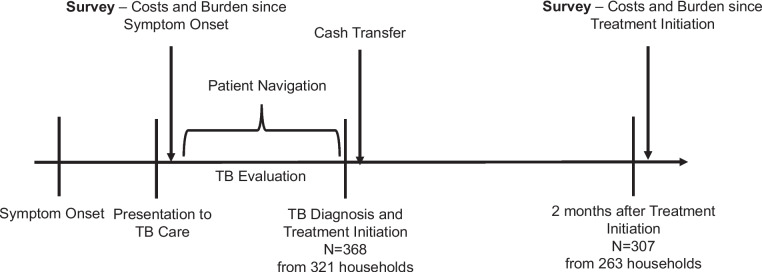
Overview of study procedures

A standardized questionnaire was administered to caregivers at TB evaluation and 2 months after diagnosis and treatment initiation (Fig. 1 and Additional file 1). We asked if the household’s finances were negatively affected by the child’s illness. We also asked if the child’s illness impacted direct medical costs, loss of income, dissaving practices, missed school and caregiver concerns of the child falling behind developmentally. Provided examples of developmental delays to caregivers included speech, learning, and motor function delays. As noted in Fig. 1, the survey at TB evaluation assessed the social and financial burden from symptom onset, while the follow-up survey assessed the social and financial burden in the 2 months since treatment initiation. We did not administer the full WHO TB patient cost survey [24], as a national costing initiative was recently conducted in Uganda [20], and our primary goal was to assess caregiver perception of social and financial burden of TB before and after the multi-component intervention.

Socioeconomic status (SES) was determined based on asset ownership, such as electronics, motor vehicles, furniture, and whether the household had working electricity. SES tertile was determined using principal components analysis (PCA) [25]. We used the first principal component, which accounted for the largest variance in the dataset, and divided the PCA score into tertiles. We generated this wealth index to measure household SES rather than using reported household income, as income is often unreliable and difficult as an indicator of relative wealth in settings like Uganda [26].

### Statistical analysis

We summarized baseline clinical characteristics of children with TB and sociodemographic characteristics of households, stratified by household SES tertile. Income and costs were presented in USD. Children in the same household can present to TB care at different times, and their caregivers may provide variable answers to the same household-level questions. For discrepant household demographic variables, we used the mean of the response for continuous variables and excluded discrepant dichotomous responses. For discrepant answers on the consequences of TB on the household, we used the mean for continuous responses and defined dichotomous answers as affirmative if at least one response in the household was “yes.” For our primary outcome, we calculated the overall proportion of households with catastrophic costs due to TB and compared it across SES tertile at baseline and two months. We utilized Kruskal–Wallis, Fisher’s exact and Chi-squared testing with significance defined as a p-value < 0.05. Differences from pre-diagnosis to two months into treatment were compared using McNemar’s test. Similar analyses were done for the secondary outcomes. Analyses were completed using Stata 16 (StataCorp, College Station, TX USA). Data is available as Additional file 2.

## Results

### Participant characteristics

We included 368 children diagnosed with drug-susceptible pulmonary TB from 321 households. Most children were under 5 years old (median 3.2 years [IQR: 1.3–6.9]) with the mother as primary caregiver and father as primary wage earner (Table 1). 194 (52.7%) children were female, 58 (16.8%) were HIV positive, and 83 (22.6%) had microbiologically confirmed TB. Children in the lowest SES tertile were more likely to be underweight. Households in the lowest SES tertile were more likely to have the primary caregiver be the same as the primary wage earner and had lower caregiver education compared to the other two SES tertiles. The average annual household income was $685.70 USD (IQR: 342.90–1371.40) and correlated with SES tertile. Twenty households (6%) had discrepant responses to at least one of the demographic and SES questions, and 24 (7.5%) had discrepant responses to at least one of the financial impact questions at baseline. At two months post-diagnosis, 9 (3.6%) had discrepant responses related to financial impact.

**Table 1:** Baseline characteristics for individuals and households with TB by socioeconomic status^a^ (*N* = 368 from 321 households)

**Child (*N* = 368)**	**Total**	**Low SES** **Tertile** **(*N* = 131)**^**b**^	**Mid-SES** **Tertile** **(*N* = 125)**^**b**^	**High SES** **Tertile** **(*N* = 112)**^**b**^	***p*-value**
Age (years)	3.2 (1.3–6.9)	3 (1.3–5.2)	3.4 (1.5–7)	3.25 (1.25–7.7)	0.26
Female	194 (52.7)	72 (55.0)	63 (50.4)	59 (52.7)	0.77
HIV positive (*N* = 345)	58 (16.8)	24 (19.8)	21 (17.8)	13 (12.3)	0.30
Underweight	107 (29.1)	54 (41.2)	30 (24.0)	23 (20.5)	0.001
Mother primary caregiver	288 (78.3)	113 (86.3)	94 (75.2)	81 (72.3)	0.02
Primary caregiver also primary wage earner	114 (31.0)	48 (36.6)	42 (34.4)	23 (20.5)	0.02
Referred from community site	183 (49.7)	64 (48.9)	67 (53.6)	52 (46.4)	0.53
Inpatient	86 (23.4)	29 (22.1)	24 (19.2)	30 (26.8)	0.15
Microbiologically confirmed TB (*n* = 369)	83 (22.6)	29 (22.1)	24 (19.2)	30 (26.8)	0.37
**Household**^**c,d**^** (*N* = 321)**	**Total**	**Low SES** **Tertile** **(*n* = 173)**	**Mid-SES Tertile** **(*n* = 161)**	**High SES** **Tertile** **(*n* = 168)**	***p*-value**
Primary Wage Earner (*n* = 320)					< 0.001
Mother	74 (23.1)	41 (36.9)	21 (18.8)	12 (12.4)	
Father	175 (54.7)	52 (46.9)	65 (58.0)	58 (59.8)	
Other	71 (22.2)	18 (16.2)	26 (23.2)	27 (27.8)	
Number in household	5 (3–6)	4 (3–6)	5 (4–6)	5 (4–7)	0.01
Others in household with TB	149 (46.6)	48 (43.2)	49 (43.8)	52 (53.6)	0.25
Annual Income (USD) (*n* = 222)	685.7 (342.9–1371.4)	342.9 (171.4–702.9)	720 (514.3–1714.3)	1028 (685.7–2742.9)	< 0.001
Maternal Education (*n* = 301)					0.02
None	66 (14.0)	21 (19.3)	19 (18.5)	10 (11.2)	
Primary School	109 (36.2)	48 (44.0)	35 (34.0)	26 (29.2)	
Secondary School	125 (41.5)	35 (32.1)	45 (43.7)	45 (50.6)	
Vocational	11 (3.7)	4 (3.7)	4 (3.9)	3 (3.4)	
University	6 (2.0)	1 (0.9)	0 (0.0)	5 (5.6)	
Paternal Education (*n* = 246)					0.001
None	42 (17.1)	25 (28.7)	9 (11.5)	8 (9.9)	
Primary School	68 (27.6)	29 (33.3)	21 (26.9)	18 (22.2)	
Secondary School	103 (41.9)	30 (34.5)	36 (46.2)	37 (45.7)	
Vocational	18 (7.3)	3 (3.5)	7 (9.0)	8 (9.9)	
University	15 (6.1)	0 (0.0)	5 (6.4)	10 (12.4)	

^a^Calculated from asset-based principal components analysis

^b^n (%) or median (IQR)

^c^Denominator is the total unless indicated

^d^Discrepant answers were averaged if continuous, or excluded if categorical

### Evaluation of multi-component intervention

Regardless of SES category, most children and households reported negative social and financial impacts prior to diagnosis and intervention (Table 2). This effect was driven primarily by the child’s TB disease, as the minority (15.2%) noted it was influenced by others in the household with TB. Households lost a median 4.9% (IQR 1.7–13.3) of annual income due to the child’s illness. This proportion was higher in households in the low and middle SES tertiles.

**Table 2:** Impact of seeking TB care for children and households

**Child**^**a**^	**Total**^**b**^	**Low SES Tertile**	**Mid-SES Tertile**	**High SES** **Tertile**	***p*-value**
Missing school (*n* = 266 children in school)	68 (25.6)	21 (23.6)	26 (26.0)	21 (27.3)	0.74
Missed 30 days or more of school	18 (6.8)	6 (6.7)	5 (5.0)	7 (9.1)	0.56
Perception that child is falling behind in development (*n* = 368)	103 (28.0)	41 (31.3)	35 (28.0)	27 (24.1)	0.83
Number of prior visits (median [IQR], *n* = 369)	3 (2–6)	3 (2–5)	3 (2–6)	3 (2–7)	0.58
Testing done at prior visits (*n* = 336)	119 (35.4)	33 (28.0)	44 (38.9)	42 (40.0)	0.11
TB testing done	39 (11.6)	12 (10.2)	13 (11.5)	14 (13.3)	0.76
Non-TB testing done	104 (31.0)	27 (22.9)	36 (31.9)	41 (39.1)	0.03
**Household**^**a,b,c,d**^	**Total**	**Low SES Tertile**	**Mid-SES Tertile**	**High SES** **Tertile**	***p*-value**
Perception that household finances negatively affected by illness (*n* = 317)	254 (80.1)	83 (76.2)	94 (83.9)	77 (80.3)	0.35
Impact includes cost of child only (*n* = 131)	111 (84.7)	32 (80.0)	35 (81.8)	44 (91.7)	0.24
Impact includes any family members with TB (*n* = 131)	20 (15.2)	8 (20.0)	8 (18.6)	4 (8.3)	0.24
Missing work (*n* = 313)	138 (44.1)	52 (47.3)	47 (42.3)	39 (42.4)	0.71
Other children in household missing school (*n* = 266)	9 (3.4)	2 (2.3)	3 (3.0)	4 (5.2)	0.56
Lost income (*n* = 319)	284 (89.0)	93 (84.6)	103 (92.0)	88 (90.7)	0.17
% annual income lost (median [IQR], *n* = 192)	4.9 (1.7–13.3)	5.2 (2.8–13.9)	5 (1.9–13.9)	2.9 (1.4–11.1)	0.03
Paying more for childcare (*n* = 310)	45 (14.5)	17 (15.7)	12 (10.9)	16 (17.4)	0.39
Paying for testing (TB or non-TB) prior to visit (*n* = 115)	90 (78.3)	23 (74.2)	34 (75.6)	33 (84.6)	0.49
At least one negative coping practice/dissavings (*n* = 313)	75 (24.0)	26 (23.9)	24 (21.6)	25 (26.7)	0.68
Taking out a loan (*n* = 311)	54 (17.4)	16 (15.0)	19 (17.1)	19 (20.4)	0.59
Selling livestock (*n* = 313)	16 (5.1)	5 (4.6)	7 (6.3)	4 (4.3)	0.77
Selling household assets (*n* = 313)	31 (9.9)	13 (11.9)	8 (7.2)	10 (10.8)	0.48
Total cost as proportion of annual income (median % [IQR], *n* = 350)	4.2 (1.5–13.8)	4.8 (1.3–12.5)	5.0 (1.6–14.6)	3.1 (1.5–11.8)	0.85
Catastrophic cost (≥ 20% annual income) (*n* = 217)	40 (18.4)	16 (18.8)	14 (19.4)	10 (16.7)	0.91

^a^Denominator is the total N of children (368) or total N of unique households (321) unless indicated

^b^Responses reported as N (%) unless indicated as median [IQR]

^c^Discrepant answers within a household were averaged if continuous, or categorized as “Yes” if at least one caregiver responded accordingly

^d^Missing data for variables attributed to if caregiver declined to answer or if question was not applicable (i.e., proportion of income lost not asked if income lost was not indicated)

After two months, we surveyed caregivers of 307 children across 263 households due for a follow-up visit. Of the 61 who did not return to follow up, 2 had withdrawn from the study (3%) and 13 (21%) died. Those without follow-up data had a higher proportion in the low SES tertile (27/61, 44%), although the majority in the low tertile (104/131, 79%) returned to follow up. Compared to the pre-diagnosis assessment, caregivers reported a limited impact of TB on the child or household finances (Table 3).

**Table 3:** Impact of TB management on children and households, two months after multi-component intervention

**Child**	**Total**^**b**^	**Low SES** **Tertile**	**Mid-SES** **Tertile**	**High SES Tertile**	***p*-value**	**Difference compared to pre-diagnosis**^**c**^** (95% CI)**
Missed school in the last 2 months (*n* = 208 children in school)	4 (1.3)	2 (3.1)	1 (1.2)	0 (0.0)	0.50	-25.4 (-31.9 to -18.8); *p* < 0.001
Perception that child is falling behind in development (*n* = 289)	0	0	0	0	-	-28.2 (-33.8 to -22.6); p < 0.001
**Household**^**a**^	**Total**	**Low SES Tertile**	**Mid-SES Tertile**	**High SES Tertile**	***p*-value**	**Difference compared to pre-diagnosis**^**3**^
Perception that household finances negatively affected by illness (*n* = 248)	12 (4.8)	4 (4.9)	3 (3.4)	5 (6.4)	0.67	-75.2 (-81.2 to-69.2); *p* < 0.001
Missed work in the last two months (*n* = 248)	4 (1.6)	2 (2.4)	1 (1.1)	1 (1.3)	0.77	-45.5 (-52.1 to -38.8); *p* < 0.001
Other children in household missed school in the last two months (*n* = 248)	1 (0.4)	0	1 (1.1)	0	0.40	-1.8 (-4.5 to 0.8); *p* = 0.22
Lost income (*n* = 247)	4 (1.6)	1 (1.2)	1 (1.1)	2 (2.6)	0.69	-87.5 (-92.0 to -83.0); *p* < 0.001
Paying more for childcare (*n* = 248)	1 (0.4)	0	0	1 (1.3)	0.34	-15.4 (-20.3 to -10.4); *p* < 0.001
At least one negative coping practice/dissavings (*n* = 248)	2 (0.8)	0	1 (1.1)	1 (1.3)	0.76	-22.5 (-28.3 to -16.8); *p* < 0.001
Taking out a loan (*n* = 248)	2 (0.8)	0	1 (1.1)	1 (1.3)	0.76	-15.7 (-20.8 to -10.6); *p* < 0.001
Sold asset (Livestock or household asset) *n* = 255)	10 (3.9)	3 (3.6)	3 (3.3)	4 (4.9)	0.85	-10.2 (-15.5 to -4.8); *p* < 0.001
Catastrophic cost (≥ 20% annual income) (*n* = 244)	1 (0.4)	1 (1.3)	0	0	0.64	-18.6 (-25.0 to -12.2); *p* < 0.001

^a^Discrepant answers within a household were averaged if continuous, or categorized as “Yes” if at least one caregiver responded accordingly

^b^Responses reported as N (%)

^c^Among those who completed both the baseline and follow up questionnaire

### Primary outcome

Prior to intervention, 18.4% (95% CI 13.7–24.0), *n* = 40 households) incurred catastrophic costs due to seeking TB care, consistent across SES tertiles. Two months after the multi-component intervention, only one household (0.4%) reported catastrophic costs (Table 3).

### Secondary outcomes

Before intervention, 80% (*n* = 254, 95% CI 75.4–84.2) of households reported that their household finances had been negatively affected during care seeking. Post-diagnosis, only 12 households (4.8%) perceived that their household finances were being negatively impacted. Similarly, 89% (*n* = 284 households, 95% CI 85.2–92.0) reported losing income, and 44% (*n* = 138 households, 95% CI 38.3–49.2) missed work to attend to their child. After intervention, only four households (1.6%) noted losing income or missing work due to their child’s illness, with a mean income loss of $8.61 USD (11.9% of annual income). The other households did not report lost income post-diagnosis. Prior to intervention, a quarter (*n* = 75 households, 24%, 95% CI 19.5–28.9) reported in engaging in at least one type of dissaving practice. At 2 months post-diagnosis, dissaving practices were uncommon, with only two households (0.8%) indicating a negative coping strategy.

More than a quarter (*n* = 103, 28%, 95% CI 23.6–32.7) of parents perceived that their child was falling behind in development, and 25% of children (95% CI 20.6–31.0) had missed school prior to TB diagnosis. At 2 months post-diagnosis, no parents perceived that their child was behind in development, and only four caregivers (1.3%) reported that their child had missed school.

## Discussion

Social protection programs for TB disease have focused on adults, and costs incurred due to personal illness. Our findings demonstrate that households face significant economic challenges when seeking TB diagnostic care for their child, and a multi-component intervention that addressed these costs and challenges could mitigate ongoing burden across SES categories. These findings support that TB-specific social protection interventions have the potential to reduce the socioeconomic burden of childhood TB on families and address the global priority to eliminate catastrophic costs due to TB.

In the process of seeking care, 80% of households noted that their child’s illness impacted their finances. Ultimately, we found that almost one in five households reported catastrophic costs. This high pre-diagnosis burden is reflective of the technical and systemic challenges that delay a diagnosis of pediatric TB [27], as children averaged three prior medical visits and over a third had prior testing. At the same time, this estimate is lower than the global catastrophic cost prevalence (48%) and the Uganda national TB costing survey (65%) [6, 20]. It is likely that our finding is an underestimate as we did not administer the entire WHO TB costing survey. However, our results are consistent with past studies that households with children who have TB face a large financial burden [17, 20], and interventions are needed to reduce catastrophic costs due to TB.

Children with TB can also have significant school disruption [12], and we found a quarter of children missed school and more than a quarter of caregivers were concerned that their child may experience developmental delays. Parents have previously cited that they are worried that their child will not perform as well as peers, be behind in their studies, and will not be prepared for exams due to TB, or that their child will face future barriers due to TB stigma [17]. In previous qualitative work, caregivers also observed detriments to the cognitive and emotional wellbeing of their child [18]. It is well-recognized with other infectious diseases that school disruption, poverty, food insecurity, malnutrition, and psychosocial stress can contribute to gaps in developmental potential and the importance of early intervention [28, 29]. For example, a social protection intervention that combined cash grants and food security improved educational and cognitive outcomes for HIV positive children in South Africa and Malawi [14]. In considering a child-sensitive social protection program for TB, our findings support linkage to services that support education and development [30].

Two months after receiving benefits, most caregivers did not report ongoing negative effects on their child or household. Although we did not have a comparison group who did not receive benefits, several national TB costing surveys including from Uganda have found that post-diagnosis costs are high [31, 32]. In other settings, the highest costs were experienced two months following treatment initiation [1], and in Uganda, the estimated post-diagnosis costs were about ten times the average monthly wage [20]. These costs are driven by direct non-medical expenses including travel, nutritional supplements and food, and in the Uganda national survey, these were higher in children than adults [4, 20]. We provided cash transfers to support direct non-medical costs, and while it primarily supported transportation, the amount provided (3–10% of annual income depending on SES tertile) likely could cover other costs. Indirect costs from loss of income were the second largest costs to caregivers of children with TB in the national survey. Support of direct medical costs and patient navigation may have helped mitigate indirect costs by allowing immediate initiation of treatment so that caregivers could return to work as soon as possible. This hypothesis is supported by the large drop in total costs after only 2 months of care, with most reporting no further loss of income or missed work. Further studies are needed to quantify the socioeconomic effects and treatment outcomes of a child-sensitive TB social protection strategy in comparison to children who received standard TB care.

In this pilot, we utilized a large cohort of children initiated on TB treatment with close follow-up. However, there were limitations that can inform additional studies. Longer follow-up is needed with a comparison group who receives standard TB care alone. For households with discrepant responses, we included any affirmative answer and may have overestimated burden, although the majority of households did not have discrepant responses. As our goal was to assess pre-diagnostic burden, we excluded children with a prior TB diagnosis. Further evaluation is needed for children and families who were either lost to follow up or had relapse or recurrence. They may have a greater social and financial burden, as we found those who did not return to follow up represented a larger proportion with low SES. The amount of cash provided per household may not be feasible to implement on a larger scale, and data from our study and national costing surveys can inform covering direct and/or indirect costs [4]. Future work could also include formal developmental screening instead of caregiver report. Returning to school is related to clinical improvement, and further studies are needed to examine how social protection interventions can improve treatment outcomes in children. This study was conducted at a tertiary care center, and more research is needed in routine care settings. Lastly, studies have shown a larger socioeconomic burden for households with multi-drug resistant (MDR) TB and HIV co-infection [18, 32], and additional work is needed to assess social protection interventions for these groups.

## Conclusions

Pediatric TB can harm a family’s finances, disrupt education and raise caregiver concern about their child’s development. From a life course perspective, these factors can increase a child’s risk of becoming an adult living in poverty, perpetuating the same challenges for the next generation [13]. It is important to mitigate these transgenerational effects through child-sensitive social protection programs [33]. We found that measures including support for direct medical costs, cash transfers for non-medical costs, and patient navigation could diminish the post-diagnostic burden for households of children with TB. Future efforts are needed to identify the optimal strategy for delivering comprehensive social protection for children and their caregivers in high TB burden settings.

### Supplementary Information

**Additional file 1.** 

**Additional file 2.** 

## Data Availability

The data analyzed during this study is included as Additional file 2.
