# Supplementary material for: The socioeconomic burden of pediatric tuberculosis and role of child-sensitive social protection
Source: BMC Public Health. 2023 Nov 25;23:2339. doi: 10.1186/s12889-023-17084-7 (PMC10675962; doi:10.1186/s12889-023-17084-7)
Supplement: Supplementary file 1 — Additional file 1. [file 12889_2023_17084_MOESM1_ESM.pdf]

Additional file 1

File format: pdf

Title of data: Survey questions at baseline and follow up

Description of data: List of questions presented to participants and caregivers during study related to socioeconomic status and burden at the baseline and follow-up visits.

SURVEY – INITIAL EVALUATION

Socioeconomic Questions

Highest level of education mother achieved:

- ◯ None
- ◯ Primary
- ◯ Secondary
- ◯ Vocational
- ◯ University
- ◯ Don't know

Highest level of education father achieved:

- ◯ None
- ◯ Primary
- ◯ Secondary
- ◯ Vocational
- ◯ University
- ◯ Don't know

Highest level of education caregiver achieved:

- ◯ None
- ◯ Primary
- ◯ Secondary
- ◯ Vocational
- ◯ University
- ◯ Don't know

How many people live in your household?	◯ 1<br>◯ 2<br>◯ 3<br>◯ 4<br>◯ 5<br>◯ 6<br>◯ 7<br>◯ 8<br>◯ 9<br>◯ 10<br>◯ 11<br>◯ 12<br>◯ 13<br>◯ 14<br>◯ 15<br>◯ 16<br>◯ 17<br>◯ 18<br>◯ 19<br>◯ 20<br>◯ 21<br>◯ 22<br>◯ 23<br>◯ 24<br>◯ 25<br>◯ 26<br>◯ 27<br>◯ 28<br>◯ 29<br>◯ 30<br>◯ 31<br>◯ 32<br>◯ 33<br>◯ 34<br>◯ 35<br>◯ 36<br>◯ 37<br>◯ 38<br>◯ 39<br>◯ 40<br>◯ 41<br>◯ 42<br>◯ 43<br>◯ 44<br>◯ 45<br>◯ 46<br>◯ 47<br>◯ 48<br>◯ 49<br>◯ 50<br>◯ Don't Know
Who is the head of household?	◯ Mother<br>◯ Father<br>◯ Grandmother<br>◯ Grandfather<br>◯ Aunt<br>◯ Uncle<br>◯ Older sibling<br>◯ Cousin<br>◯ Other

If other, who is the head of household? ______________________

Who is the primary wager earner in the household?

- ◯ Mother
- ◯ Father
- ◯ Grandmother
- ◯ Grandfather
- ◯ Aunt
- ◯ Uncle
- ◯ Older sibling
- ◯ Cousin
- ◯ Other

If other, who is the primary wage earner in the household? ______________________

How much total income does the household earn each month? ______________________ (In Uganda Shillings (UGX))

Does the child's primary care giver earn income for the household?

- ◯ Yes
- ◯ No

How much income does the primary caregiver earn for the household each month? ______________________ (In Uganda Shillings (UGX))

In your household, do you have:

	Yes	No	Not Applicable	Don't know
Electricity Television	◯	◯	◯	◯
Motor cycle or scooter	◯	◯	◯	◯
Wardrobe	◯	◯	◯	◯
CD/DVD Player	◯	◯	◯	◯

In general, do you feel that the household's finances have been negatively affected because of the child's illness?

- ◯ Yes
- ◯ No

In particular, has the household been affected in any of the following ways because of the child's illness?

	Yes	No	Not Applicable	Don't know
The household lost income	◯	◯	◯	◯
The child had to miss school	◯	◯	◯	◯
Other children in the household had to miss school	◯	◯	◯	◯

The household had to sell livestock	◯	◯	◯	◯
The household had to take out a loan	◯	◯	◯	◯
The household had to sell other household assets like furniture, equipment, transportation	◯	◯	◯	◯
The child is falling behind in development (learning to speak, move, understand or interact with others, etc)	◯	◯	◯	◯
A member of the household had to miss work	◯	◯	◯	◯
The household had to pay more for childcare	◯	◯	◯	◯

Do any other family or household members have TB?

◯ Yes
◯ No

Does the impact you describe include the cost of caring for another family member with TB (or child only)?

◯ Child only
◯ Everyone with TB
◯ Not applicable

How much household income was lost because of the child's illness? ______________________

How many days of school has the child missed due to this illness? ______________________

How many days of school have other children in the household missed due to this illness? ______________________

How much money was taken out as a loan due to the child's illness? ______________________

How many days of work have household members had to miss because of the child's illness? ______________________

Approximately how much have you spent on childcare (beyond usual costs) due to the child's illness? ______________________

How many clinic visits did the child have for this illness BEFORE today? ______________________

Was the child tested for TB at previous visits?

◯ Yes No
◯

Did you have to pay for TB tests at previous visits?

Yes No

How much did you pay for the child's TB tests? ______________________

Did the child receive tests for NON-TB illnesses at previous visits?	◯ Yes<br>◯ No
Did you have to pay for any NON-TB tests at previous visits?	◯ Yes<br>◯ No
How much did you pay for the child's NON-TB tests?	________________________

SURVEY – 2 MONTH EVALUATION

Socioeconomic Questions

Who is the head of household?

- ◯ Mother
- ◯ Father
- ◯ Grandmother
- ◯ Grandfather
- ◯ Aunt
- ◯ Uncle
- ◯ Older sibling
- ◯ Cousin
- ◯ Other

If other, who is the head of household?

____

Who is the primary wager earner in the household?	◯ Mother<br>◯ Father<br>◯ Grandmother<br>◯ Grandfather<br>◯ Aunt<br>◯ Uncle<br>◯ Older sibling<br>◯ Cousin
If other, who is the primary wage earner in the household?	______________________
How much total income does the household earn each month?	______________________<br>(In Uganda Shillings (UGX))
Who is the primary caregiver for the child?	◯ Mother<br>◯ Father<br>◯ Grandmother<br>◯ Grandfather<br>◯ Aunt<br>◯ Uncle<br>◯ Older sibling<br>◯ Cousin
If other, who is the primary caregiver for the child?	______________________
Does the child's primary care giver earn income for the household?	◯ Yes<br>◯ No
How much income does the primary caregiver earn for the household each month?	______________________<br>(In Uganda Shillings (UGX))

In your household, do you have:

	Yes	No	Don't know
Electricity Television	◯	◯	◯
Motor cycle or scooter	◯	◯	◯
Wardrobe	◯	◯	◯
CD/DVD Player	◯	◯	◯

Since your initial visit with us, has the household been affected in any of the following ways because of the child's illness or its treatment?

	Yes	No	Don't Know
The household's finances have been affected	○	○	○
The household lost income	○	○	○
The child had to miss school			○
Other children in the household had to miss school	○	○	○
The household had to sell livestock	○	○	○
The household had to take out a loan	○	○	○
The household had to sell other household assets like furniture, equipment, transportation	○	○	○
The child is falling behind developmentally	○	○	○
A member of the household had to miss work	○	○	○
The household had to pay more for childcare	○	○	○

How much household income was lost because of the child's illness? ______________________________

How many days of school has the child missed due to this illness? ______________________________

How many days of school have other children in the household missed due to this illness? __________

How much money was taken out as a loan due to the child's illness? ______________________________

How many days of work have household members had to miss because of the child's illness? ________

Approximately how much have you spent on childcare (beyond usual costs) due to the child's illness? ______________________________
